# Construction of a high-density genetic map using specific-locus amplified fragments in sorghum

**DOI:** 10.1186/s12864-016-3430-7

**Published:** 2017-01-07

**Authors:** Guisu Ji, Qingjiang Zhang, Ruiheng Du, Peng Lv, Xue Ma, Shu Fan, Suying Li, Shenglin Hou, Yucui Han, Guoqing Liu

**Affiliations:** 1 Institute of Millet Crops, Hebei Academy of Agricultural & Forestry Sciences/Hebei Branch of China National Sorghum Improvement Center, Shijiazhuang, 050035 China; 2 Institute of Cereal and Oil Crops, Hebei Academy of Agricultural & Forestry Sciences, Shijiazhuang, 050035 China; 3 Biomarker Technologies Corporation, Beijing, 101300 China

**Keywords:** *Sorghum bicolor*, High-throughput sequencing, SLAF marker, Linkage map construction

## Abstract

**Background:**

Sorghum is mainly used as a human food and beverage source, playing an important role in the production of ethanol and other bio-industrial products. Thus it is regarded as a model crop for energy plants. Genetic map construction is the foundation for marker-assisted selection and gene cloning. So far several sorghum linkage maps have been reported using different kinds of molecular markers. However marker numbers and chromosome coverage are limited. As a result, it is difficult to get consistent results and the maps are hard to unify. In the present study, the genomes of 130 individuals consisting an F_2_ population together with their parents were surveyed using a high-throughput sequencing technique. A high-density linkage map was constructed using specific-locus amplified fragments (SLAF) markers. This map can provide information and serve as a reference for effective gene exploration, and for marker assisted-breeding program.

**Results:**

A high-throughput sequencing method was adopted to screen SLAF markers with 130 F_2_ individuals from a cross between a grain sorghum variety, J204, and a sweet sorghum variety, Keter. In the present study, 52,928 suitable SLAF markers out of 43,528,021 pair-end reads were chosen to conduct genetic map construction, 12.0% of which were polymorphic. Among the 6353 polymorphic SLAF markers, 5829 (91.8%) were successfully genotyped in the F_2_ mapping population. Finally 2246 SLAF markers were obtained to construct a high-density genetic linkage map. The total distance of linkage map covering all 10 chromosomes was 2158.1 cM. The largest gap on each chromosome was 10.2 cM on average. The proportion of gaps less than and/or equal to 5.0 cM was averagely 98.1%. The markers on each chromosome ranged from 123 (chromosome 9) to 315 (chromosome 4) with a mean value of 224.6, the distance between adjacent markers ranged from 0.6 (chromosome 10) to 1.3 cM (chromosome 9) with an average distance of only 0.98 cM.

**Conclusion:**

A high density sorghum genetic map was constructed in this study. The total length was 2158.1 cM covering all 10 chromosomes with a total number of 2246 SLAF markers. The construction of this map can provide detailed information for accurate gene localization and cloning and application of marker-assisted breeding.

**Electronic supplementary material:**

The online version of this article (doi:10.1186/s12864-016-3430-7) contains supplementary material, which is available to authorized users.

## Background

Sorghum (*Sorghum bicolor*) is one of the five dominant crops in the world including corn (*Zea mays*), wheat (*Triticum aestivum*), rice (*Oryza sativa*) and barley (*Hordeum vulgare*) (http://www.fao.org). With the advantages of high yielding, good adaptability, drought, salt and alkali tolerance, it is one of the most valuable energy crops for the future [1, 2]. Sorghum is a typical C_4_ crop and mainly used as a human food and beverage source. Sorghum grain is the main ingredient of top-grade alcohols and its stem can be used as fodder. Both grain and stem including all plant are stock for bioethanol and other bio-industrial products.

The genome size of sorghum (750 Mb) is 3–4 times smaller than corn, thus it was regarded as a diploid model crop for energy plants like polyploidy sugarcane and *Miscanthus* [3, 4]. A genetic map is a foundation for quantitative and qualitative gene mapping and cloning, and plays a key role in marker-assisted breeding program. High-density genetic maps of sorghum can be used for genome comparison, useful gene mining and gene mapping. The genes for disease and insect-resistance, stress tolerance, sugar concentrations and biological yield can be identified by comparing homology in different plant species, and they can also be located on chromosomes by mapping, which lays a foundation for gene cloning and application. High-density genetic mapping has great importance in increasing statistical power and precision of detecting genes and QTLs.

Genetic map construction of sorghum began in 1990s. The early linkage maps of sorghum were constructed mainly by using labor-intensive or dominant markers such as RFLP (Restriction fragment length polymorphism), AFLP (Amplified fragment length polymorphism) and RAPD (Random amplified polymorphic DNA) [5–10]. These maps have played important role in sorghum gene (QTL) mapping, comparative genomics and genetics studies. However, these genetic marker systems have limited marker numbers, dominant expression, and not repeatable in different maps. More informative marker types can effectively overcome the disadvantages mentioned above are required. Due to the quick development of sequencing and genotyping technologies, simple sequence repeat (SSR) with features of high reproducibility, co-dominant inheritance, multi-allelic variation and abundance in the genome, have replaced dominant markers for constructing linkage maps. SSR markers were first used for polymorphism detecting and linkage group identification [11, 12], then were used to construct sorghum genetic maps with the development of a large amount of SSR markers [13, 14]. Several linkage maps with SSR markers or mainly based on SSR markers have been developed and have been using in sorghum gene (QTL) mapping, genome evolution, molecular genetics and marker-assisted breeding [4, 15–18].

However, the above technologies such as RAPD, RFLP, AFLP and SSR to determine genetic fingerprints have limitations to cover full genome which requires the identification of a large number of polymorphic markers. With these technologies this is a step by step approach that is labor intensive and plagued by process variation. Diversity Arrays Technology (DArT) was initially used to detect a large number of genetic differences between plant and animal varieties. Recently this technology was introduced for sorghum map construction. DArT markers were integrated into a sorghum consensus map which consisted of a total of 1997 markers mapped to 2029 unique loci (1190 DArT loci and 839 other loci) spanning 1603.5 cM and with an average marker density of 1 marker/0.79 cM [19].

Great progress has been made in the sequencing technologies and bioinformatics at an exponentially reduced cost, which led to a revolution in the field of genotyping technologies. Restriction associated DNA sequencing (RAD-seq) and genotyping by sequencing (GBS) have emerged as powerful genotyping platforms, which are capable of identifying, sequencing, and genotyping thousands of markers across almost any genome of interest and number of individuals in a population [20]. The next generation sequencing can directly determine differences in DNA sequence with high accuracy, thus it has been widely used for plant and animal genetic analysis. SLAF (specific-locus amplified fragments) markers, which has been used for genetic investigation, have the properties of being present in large amount, being evenly distributed and avoiding repeated sequences [21]. These markers have been used for crop genetic analysis such as sesame, millet, rice and soybean [22–26], especially in the applications of high-density genetic map construction and functional genes verification. Exploiting this approach to scan the whole sorghum genome has great importance for high density marker development and gene mining for sorghum breeding.

The purpose of this study is to construct a high-density linkage map with SNPs through next generation sequencing technology. The map can provide information and serve as a reference for effective gene exploration and lay a foundation for marker assisted breeding. Further, it can benefit the development of biological energy resources.

## Results

### Parents and F_2_ population for map construction

An F_2_ population consisting of 130 individuals from a cross of Keter × J204 was used for genetic map construction. The maternal parent is a sweet sorghum variety and the paternal parent is a grain sorghum variety. There have been great differences in phenotypic characters between the two parents, such as plant height, heading time, seed coat color, etc. Therefore, their offspring will have considerable variations which are good for polymorphic marker screening and linkage group construction.

### Marker identification

**Fig. 1:**
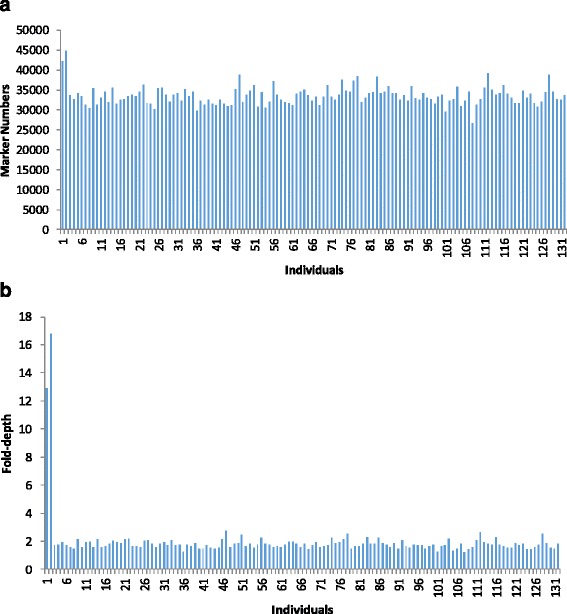
The SLAF number and the sequencing-depth in the parents and F_2_ individuals. **a** Number of SLAF markers. **b** Sequencing-depth of SLAF markers. The X-axis in (**a**) and (**b**) indicates individuals including maternal parent (Keter, designated as 1), paternal parent (J204, designated as 2) and 130 individuals from the F_2_ population. The Y-axis indicates the number of reads in (**a**) and the sequencing-depth in (**b**)

**Fig. 2:**
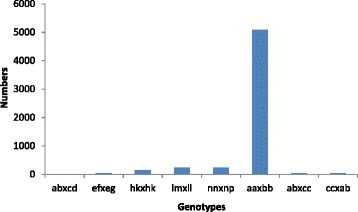
The marker numbers in different segregation patterns. The X-axis indicates different segregation patterns. The Y-axis indicates the SLAF number in each pattern

The SLAF number and sequencing depth identified in the parents and their offspring were plotted in Fig. 1a. The SLAF marker number in paternal and maternal parents was 44,895 and 42,100, respectively. The sequencing depth on average was 16.8-fold in paternal parent and 12.9-fold in maternal parent. The SLAF numbers for each F_2_ individual ranged from 26,737 to 39,291 with an average of 33,445.1. The sequencing depth shifted from 2.2 to 3.7-fold with an average of 2.8-fold (Fig. 1b). Among the 52,928 (Additional file 1) qualified SLAF markers, 6353 were polymorphic with a polymorphism rate of only 12.0% (Table 1). Of the 6353 polymorphic SLAF markers, 5829 (91.8%) were classified into eight segregation patterns (Fig. 2). Among them 5093 (87.4%) markers fell into segregation pattern aa × bb. Because individuals in the F_2_ population which was obtained by selfing the F_1_ of a cross between two fully homozygous parents showed this genotype, only the aa × bb segregation pattern in the F_2_ population was used to construct the genetic map. Finally 2,246 (Additional file 2) markers were assigned onto linkage groups.

**Table 1:** SLAF marker identification

Type	SLAF number	Ratio (%)
Polymorphisms	6353	12.0
Non-polymorphisms	46575	88.0
Total	52928	100.0

The average sequencing depths were 29.3-fold in the parents and 3.3-fold in the offsprings on linked markers (Table 2). This integrity and depth of markers were enough to guarantee the accuracy for genetic map construction [21].

**Table 2:** The sequencing depth of assigned makers in the parents and F_2_ population

Name	Marker number	Total depth	Average depth
Keter	2246	59620	26.5
J204	2246	71689	31.9
Offspring	1971	7296	3.3

### Linkage map construction

All the 2246 assigned markers were grouped to 10 chromosomes, the linear alignments of markers on chromosomes were built by the genetic distances between adjacent markers. Finally the 2246 markers were assigned onto the genetic map with a total length of 2158.1 cM and average distance between markers of 0.98 cM. The degree of linkage between markers was reflected by Gap less than and/or equal to 5.0 cM (Gap < = 5) ranging between 96.3% and 100.0% with an average value of 98.1%. The largest gap on chromosome 7 is 15.7 cM. On average 224.6 markers were assigned on each chromosome with a length of 215.8 cM (Table 3).

**Table 3:** Map information based on high quality SLAFs obtained from population sequencing

Linkage groups	Marker types and numbers				Total distance (cM)	Average distance between markers (cM)	Largest gap (cM)	Gap < =5 (%)
	Total	SNP only	InDel only	SNP & InDel				
1	200	199	1	0	238.7	1.20	14.1	97.5
2	292	290	0	2	287.9	0.99	8.5	99.0
3	250	249	0	1	286.7	1.15	7.4	98.4
4	315	314	0	1	300.4	0.96	13.7	98.1
5	218	217	1	0	183.7	0.85	9.4	97.2
6	216	213	1	2	175.0	0.81	10.7	98.1
7	217	217	0	0	266.3	1.23	15.7	96.3
8	189	189	0	0	134.6	0.72	8.6	98.9
9	123	123	0	0	152.2	1.25	4.3	100.0
10	226	226	0	0	132.8	0.59	9.7	97. 8
Total	2246	2237	3	6	2158.1	0.98	10.2	98.1

Among the 2246 markers, 315 were assigned on chromosome 4 which was the largest in the ten chromosomes. The total length was 300.4 cM with an average distance of only 0.96 cM between adjacent markers. A large gap of 13.7 cM was located between 245.6 to 259.3 cM, the gap < = 5 ratio was 98.1%. The fewest markers (123) were on chromosome 9, which was 152.2 cM in length with an average distance of 1.3 cM between adjacent markers. A large gap of 4.3 cM was located at the end of the chromosome. The gap < = 5 ratio was 100.0% which indicates the good quality of marker assignment (Table 3).

### Map evaluation

Three types of markers were assigned to the genetic map including 2237 ‘SNP_only’, 3 ‘InDel_only’, and 6 ‘SNP&InDel’ markers. ‘SNP_only’ was the predominant marker type accounting for 99.6% of the markers. ‘InDel_only’ markers were assigned on chromosomes 1, 5 and 6, respectively. While 6 ‘SNP&InDel’ markers were assigned on chromosomes 2, 3, 4 and 6, respectively (Table 3).

Of the all 2237 SNP markers, most were transition type SNPs with R (G/A) and Y (T/C) types accounting for 32.8% and 32.7%, respectively. The other four SNP types were transversions including S (G/C), M (A/C), K (G/T), and W (A/T) with percentages of 9.1, 8.4, 8.3 and 8.7 of all SNPs, respectively (Table 4).

**Table 4:** Different SNP types in the linkage group

SNP types	Number	Ratio (%)
R(G/A)	915	32.8
Y(T/C)	914	32.7
S(G/C)	255	9.1
W(A/T)	243	8.7
K(G/T)	231	8.3
M(A/C)	234	8.4
Total	2792	100.0

**Fig. 3:**
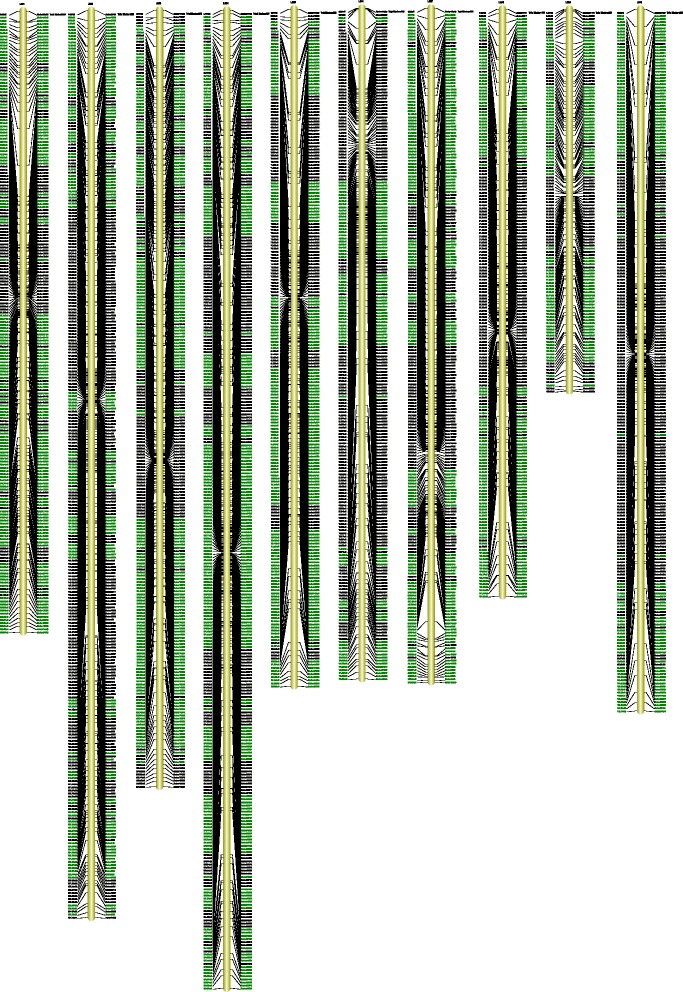
Ten linkage groups of sorghum from a cross of Keter × J204. SLAF marker names and their locations are listed on the right and left sides of the axis. Segregation distortion markers on the map are highlighted in *green*

Markers that showed significant (*χ* ^2^, *p* < 0.05) segregation distortion (1192 in total) were finally assigned onto the map (Fig. 3) and most of them were clustered at the two ends of chromosomes and some located at chromosome centers such as chromosome 4 (Table 5, Fig. 3). More than half (53.1%) of the assigned markers showed significant (*p* < 0.05) segregation distortion which distributed on each chromosome. The largest chromosome (chr. 4) had the highest percentage of segregation distortion markers (15.8%) and the smallest chromosome (Chr. 9) had the lowest percentage of segregation distortion markers (6.0%). All the distorted markers clustered into 98 segregation distortion regions (SDRs) which distributed on each chromosome. Similarly 14 SDRs were found on chr 4 and 6 on chr 9 (Table 5). Among the three different marker types assigned to the final map, no one marker type was observed to show a particular tendency for skewness. Besides ‘SNP-only’ markers, one out of 3 ‘InDel-only’ and 4 out of 6 ‘SNP&InDel’ markers showed segregation distortion, respectively.

**Table 5:** Description on segregation distortion markers

LGs	Total marker		Segregation distortion marker		*χ* ^2^	*P*	Distorted markers on each LG (%)	SDRs
	No.	%	No.	%				
1	200	8.9	132	11.1	20.644	0.005	71.5	12
2	292	13.0	112	9.4	15.147	0.008	38.4	13
3	250	11.1	156	13.1	19.837	0.007	62.0	13
4	315	14.0	188	15.8	19.416	0.006	59.7	14
5	218	9.7	140	11.7	20.009	0.006	64.2	7
6	216	9.6	74	6.2	18.813	0.007	34.3	7
7	217	9.7	131	11.0	21.687	0.006	60.4	10
8	189	8.4	101	8.5	21.148	0.004	53.4	8
9	123	5.5	72	6.0	19.949	0.005	58.5	6
10	226	10.1	86	7.2	20.354	0.007	44.3	5
Total	2246		1192					98

## Discussion

In the present study an F_2_ mapping population from a cross between a sweet and a grain sorghum variety was employed to construct a sorghum linkage map. The great character variations between the two parents benefited the marker polymorphism discovery. The high-throughput sequencing technology used in the present study has greatly enhanced the identification and guaranteed the quantity and quality of markers. Therefore a high density genetic map was successfully constructed. Some existing sorghum maps are unsatisfactory for gene identification because of lacking adequate markers from the whole genome, and broken chromosome segments. In the present study a dense genetic map was generated in which the whole sorghum genome sequence was surveyed, high quality markers were identified and uniformly distributed on 10 chromosomes. Each chromosome contains 123–318 markers and its length ranged from 132.8 to 300.4 cM. This high density SNP-based linkage map for sorghum can serve as a reference map for cultivated sorghum species and will be useful in genetic mapping.

DNA marker distribution is not random with some clear marker-dense regions and some marker deserts. In the present map, marker deserts (gaps) were observed with varied sizes. Most (97.0%) of the gaps on every linkage group are less than and/or equal to 5.0 cM. In total only 11 gaps larger than 5.0 cM were detected in all chromosomes except chr 9 suggesting that such gaps are not restricted to a particular chromosome. Gaps larger than 10.0 cM were found on chromosomes 1, 4, 6 and 7. The longest one was 15.7 cM on the distal end of chr 7. The presence of these gaps may have negative effects on the application of mapped DNA markers, for example, genomic regions that lack DNA markers will make detection of quantitative trait loci (QTL) difficult [27]. Therefore, more comparable markers between different sorghum maps are needed to fill in the gaps to obtain a more complete coverage of the sorghum genome.

Segregation distortion is a common phenomenon in which the genotypic frequency of a marker deviates from a typical Mendelian ratio. Previous studies have showed that a large number of segregation distortions and SDRs occur in many species, such as maize [28], barley [29] potato [30], sesame [24], peanut [31] and sorghum [3, 19]. The genetic basis of segregation distortion is still under debate, and gametophyte and/or zygotic selection and chromosomal rearrangements may be the main cause of this phenomenon [3]. However, some studies found segregation distortion in a non-random and consistent distribution pattern suggested that distorted segregation is due to the elimination of gametes or zygotes by a lethal factor located in a neighboring region of the marker [19]. On a sorghum consensus map, chr 1 has the highest proportion of chromosomal regions associated with skewed segregation (67%). Two other chromosomes (chr 4 and chr 8) also have over 50% of the chromosomal regions associated with skewed segregation (51.6% and 54.1%, respectively) [19]. In the present study, an F_2_ mapping population was employed to construct a linkage map, among the 2246 assigned markers, 1192 markers (53.1%) showed significant segregation distortion. All the skewed markers clustered into segregation distortion regions. Although it is not exactly the same, chr 1 has the highest proportion (71.5%) of skewed markers and chr 4 has the biggest number (14) of SDRs in this final map, which indicates that there may be similar mechanism of skewed segregation phenomenon between the two studies. Further, studies have proved that the presence of segregation distortion markers will not affect the use of linkage maps for applications such as QTL mapping [32, 33].

Genomic approaches such as high-throughput sequencing and large-scale genotyping technologies have been used in genetic linkage mapping. The SLAF-seq method provided significant advantages to generate enough polymorphic markers for high-density genetic map construction. The high density map is sufficient to ensure adequate polymorphic marker coverage in regions of interest and can be used as a reference map for sorghum genetic studies.

## Conclusions

A high density sorghum map was constructed in this study by employing SLAF markers developed from high-throughput sequencing technology. The total map length is 2158.1 cM covering sorghum 10 chromosomes with a total of 2246 SLAF markers. The construction of this map can provide detailed information for gene localization, cloning and application of marker-assisted breeding.

## Methods

### Plant materials

An F_2_ mapping population derived from a cross of sweet sorghum Keter and grain sorghum J204 (a variation line of J14859 from the USA) was employed to construct a linkage map. The parents and F_2_ individuals were planted in the Experiment Station, Institute of Millet Crops, Shijiazhuang, China in the year 2012, the heading date was recorded and the heads were bagged prior to anthesis to prevent out crossing contamination and allowed to self-fertilize. Five plants of each parent and 130 F_2_ individuals were phenotyped.

### DNA extraction

DNA was extracted from fresh leaf tissue following the modified CTAB protocol [34]. DNA concentration was adjusted to be in the range of 50–100 ng μl^−1^.

### SLAF library construction and high-throughput sequencing

An improved SLAF-seq strategy was used in this study. Firstly, sorghum genome was used as reference to design the experiments for marker discovery by simulating *in silico,* different enzymes were adopted to produce a lot of markers. Next, predesigned scheme was used to construct the SLAF library. Enzyme *MseI* (New England Biolabs, NEB, (USA)) was adopted for the F_2_ population. After digested the genomic DNA, a single nucleotide (A) overhang was added to the digested fragments using Klenow Fragment (3´ → 5´ exon) (NEB) and dATP at 37 °C. Duplex tag-labeled sequencing adapters (PAGE-purified, Life Technologies, USA) were then ligated to the A-tailed fragments using T_4_ DNA ligase. Diluted restriction-ligation DNA samples were used to performe the polymerase chain reaction (PCR): dNTP, Q5 high-fidelity DNA polymerase and PCR primers (Forward primer: 5’-AATGATACGGCGACCACCGA-3’, reverse primer: 5’-CAAGCAGAAGACGGCATACG-3’) (PAGE-purified, Life Technologies). Then purified and pooled the PCR products by agencourt AMPure XP beads (Beckman Coulter, High Wycombe, UK). 2% agarose gel electrophoresis was used to separate pooled samples. Took the fragments ranged from 380 to 410 base pairs (with indexes and adaptors) in size from the gel and excised and purified using a QIAquick gel extraction kit (Qiagen, Hilden, Germany). After diluted, the pair-end sequencing (Each end 125 bp) was performed on an Illumina HiSeq 2500 system (Illumina, Inc; San Diego, CA, USA) according to the manufacturer’s recommendations.

### Sequence data grouping and genotyping

Procedures described by Sun et al. [21] was adopted to SLAF marker identification and genotyping. After the low-quality reads (quality score < 20e) were filtered out, the SLAF pair-end reads with clear index information were clustered based on sequence similarity (BLAT) [35, 36] (−tileSize = 10 –step Size = 5). Sequences with over 95% identity were grouped in one SLAF locus. Single nucleotide polymorphism (SNP) loci of each SLAF locus were then detected between parents, and SLAFs with more than 3 SNPs were filtered out firstly. Alleles were defined in each SLAF using the minor allele frequency (MAF) evaluation.

Groups containing more than four tags were filtered out as repetitive SLAFs for a diploid species like sorghum that one locus contains at most four SLAF tags. The low-depth SLAFs which with a less than 2.20 sequence depth were filtered out and the SLAFs with 2, 3, or 4 tags were identified as polymorphic SLAFs which were the potential markers. Polymorphic markers were classified into eight segregation patterns (ab × cd, ef × eg, hk × hk, lm × ll, nn × np, aa × bb, ab × cc and cc × ab). Because individuals in the F_2_ population which was obtained by selfing the F_1_ of a cross between two fully homozygous parents showed segregation pattern aa × bb, SLAF markers showing other segregation patterns which caused by parental heterozygosity were unsuited genotypes and filtered out for mapping. The SLAFs with low integrity percentage and seriously segregation distortion were filtered out too. Then SLAF markers which segregation patterns were aa × bb was used only for linkage construction. The average sequence depth of SLAF markers were greater than 20-fold in parents and 3-fold greater in progeny, the integrity percentage both in the progeny and in the parents were 80% above.

Bayesian method was used to score the genotype and ensure its quality. First, the coverage of each allele and the number of single nucleotide polymorphism were used to calculate the posteriori conditional probability. Next, the qualified markers for subsequent analysis were selected from the probability translated from genotyping quality score [37]. Low-quality markers and the worse marker or individual were deleted during the dynamic process, the process stopped when the average genotype quality scores of all SLAF markers reached the cutoff value.

The following criteria was adopted to filter the high-quality SLAF markers for the genetic mapping. 1) The average sequence depths should be more then 3-fold in each progeny and more than 29-fold in the parents. 2) Markers with more than 30% missing data were filtered. 3) The *chi-square* test was performed to examine the segregation distortion. Markers with significant segregation distortion (*p* < 0.05) were initially excluded from the map construction and were then added later as accessory markers.

### Linkage map construction

According to the locations on the genome, marker loci were partitioned primarily into linkage groups (LGs). Markers with MLOD scores < 5 were filtered, and then, the modified logarithm of odds (MLOD) scores between markers were calculated to further confirm the robustness of markers for each LGs. To ensure efficient construction of the high-density and high-quality map, a newly developed high map strategy was utilized to order the SLAF markers and correct genotyping errors within LGs [38]. Firstly, recombinant frequencies and LOD scores were calculated by two-point analysis, which were applied to infer linkage phases. Then, enhanced Gibbs sampling, spatial sampling and simulated annealing algorithms were combined to conduct an iterative process of marker ordering [38, 39]. Summation of adjacent recombination fractions was calculated as illustrated by Liu et al. [40].

While a number of successive steps, the annealing system continued until the newly generated map order is rejected. Blocked Gibbs sampling was employed to estimate multipoint recombination frequencies of the parents after the optimal map order of sample markers were obtained. The updated recombination frequencies were used to integrate the parental maps and optimize the map order in the next cycle of simulated annealing. Once a stable map order was obtained after 3–4 cycles, the next map building would be turned round. The unmapped markers was selected and added to the previous sample. The mapping algorithm repeats until all the markers were mapped appropriately. The error correction strategy of SMOOTH was then conducted according to parental contribution of genotypes [41] and a k-nearest neighbor algorithm was applied to impute missing genotypes [42]. Skewed markers were then added into this map by applying a multipoint method of maximum likelihood [43]. Map distances were estimated using the Kosambi mapping function [43].

## Additional files

Additional file 1: Details of assigned marker sequences. (TXT 1116 kb)
Additional file 2: Details of F_2_ individual genotypes. (GENOTYPE 896 kb)

## Abbreviations

AFLP: Amplified fragment length polymorphism
DArT: Diversity arrays technology
InDel: Insert and deletion
LOD: Logarithm of odds
PCR: Polymerase chain reaction
QTL: Quantitative trait locus
RAPD: Random amplified polymorphic DNA
RFLP: Restriction fragment length polymorphism
RIL: Recombinant inbred line
SLAF: Specific length amplified fragments
SNP: Single nucleotide polymorphisms
